# Attentional Mechanisms during the Performance of a Subsecond Timing Task

**DOI:** 10.1371/journal.pone.0158508

**Published:** 2016-07-28

**Authors:** Anna L. Toscano-Zapién, Daniel Velázquez-López, David N. Velázquez-Martínez

**Affiliations:** 1 Departamento de Psicofisiologia, Facultad de Psicología, Universidad Nacional Autónoma de México, D.F. México, 04510, México; 2 Departamento de Matemáticas, Facultad de Ciencias, Universidad Nacional Autónoma de México, D.F. México, 04510, México; Duke University, UNITED STATES

## Abstract

There is evidence that timing processes in the suprasecond scale are modulated by attentional mechanisms; in addition, some studies have shown that attentional mechanisms also affect timing in the subsecond scale. Our aim was to study eye movements and pupil diameter during a temporal bisection task in the subsecond range. Subjects were trained to discriminate anchor intervals of 200 or 800 msec, and were then confronted with intermediate durations. Eye movements revealed that subjects used different cognitive strategies during the bisection timing task. When the stimulus to be timed appeared randomly at a central or 4 peripheral positions on a screen, some subjects choose to maintain their gaze toward the central area while other followed the peripheral placement of the stimulus; some others subjects used both strategies. The time of subjective equality did not differ between subjects who employed different attentional mechanisms. However, differences emerged in the timing variance and attentional indexes (time taken to initial fixation, latency to respond, pupil dilatation and duration and number of fixations to stimulus areas). Timing in the subsecond range seems invariant despite the use of different attentional strategies. Future research should determine whether the selection of attentional mechanisms is related to particular timing tasks or instructions or whether it represents idiosyncratic cognitive “styles”.

## Introduction

Time estimation is an essential process that allows organisms to adapt to their environment. Diverse models have emerged to explain timing. One of the first models [1] to account for timing postulated a pacemaker that sends pulses to a cognitive counter that in turn sends them to a storage mechanism; thereafter, a cognitive comparator decides if the count (or distribution) in working memory is sufficiently similar to those stored previously (reference memory) to initiate a response. Additional assumptions about the distribution of pulses from the pacemaker and the observation that the ratio of the absolute interval criteria to the standard deviation of temporal estimates tends to be constant, led to the formulation of the influential model known as Scalar Expectancy Theory (SET) [2–4]. Other cognitive models also used the pacemaker assumption, the most influential being the attentional gate model [5]. Also, there are cognitive models that do not use a pacemaker assumption [6, 7]. A fundamental distinction between the processing of time intervals below and above 1 sec has been proposed: a more ‘‘automatic” system for timing in the millisecond range, computed by the cerebellum and striatum, and a more ‘‘cognitive” system for timing in the seconds to minutes range, computed by fronto-striatal circuits (which also support working memory functions) [7–9].

Attention has been conceived as a cognitive process that allows an organism to focus selectively on some features of stimuli while excluding others [10]; such process have been invoked to account for the observation that organisms do not always produce the same response to the same stimulus in a constant environment [11]. When subjects are required to perform a non-temporal task simultaneously with a timing task, perceived time is shortened and the accuracy of temporal estimation deteriorates as more attentional resources are diverted from the temporal task [12–15]. The interference effect, resource allocation or time-sharing hypothesis refers to such disruption in timing; according to this hypothesis, performance of the non-temporal task draws attentional and/or memory resources away from performance of the temporal task, and thereby impairs time estimation [16–19]. Diverse tasks have been shown to impair time estimation and/or time production; for example, categorization or discrimination of the intensity of visual or auditory signals [14, 20], visual search or mental arithmetic [12], proofreading [21], letter reading [16], increases in memory load [22], and many more. In most of the cases mentioned, the interference task and the interval to be timed lasted for several seconds or minutes; for example, intervals of 1 to 25 minutes have been used in the ‘thinking aloud’ paradigm [23]. Timing of very short intervals may be less susceptible to disruption; for example, it has been found that estimation of the durations of auditory signals in the range of 50 msec was unaffected while durations of 500 msec or longer were influenced by the cognitive load of the concurrent task [24]. It was suggested that temporal processing in the millisecond range is of a highly perceptual nature and benefits from automatic processing and is largely independent of working memory and/or attentional allocation, whereas temporal processing of time intervals longer than 1 s is mainly cognitively mediated and susceptible to attentional manipulations [7–9, 25]. However, several studies have demonstrated that performance of a concurrent task draws attentional resources from the timing task in the subseconds range. For example, attentional effects have be found during the concurrent performance of a time reproduction and a reaction task [20],and during a production task in a range from 250 to 490 msec, [26]; also duration (200 to 1200 msec) discrimination was affected when attending to pitch [27], demonstrating attentional effects on timing within the subsecond range.

A useful distinction [13] that predicts the magnitude of the interference effect is that between retrospective timing (where subjects do not have a prior warning that a timing judgment will be required) and prospective timing (in which subjects are forewarned that judgments of time will be asked). Estimations of time are reduced in prospective conditions but the interference effect is reduced in retrospective conditions [15, 28]. To explain these findings, Block and Gruber [29] suggested a preponderance of attentional processes to timing in the prospective paradigm and a preponderance of memory for events and contextual changes in the retrospective paradigm. Early versions of timing models did not accommodate the participation of attentional mechanisms, but the interference effect has led to the incorporation of attention in most current models of timing. Models based on the assumption of the pacemaker had suggested that attention modulates the rate of the pacemaker through arousal [30, 31], switch [32, 33] or gating [29, 34] mechanisms, while more cognitively oriented models suggested that attention affects memory context [6, 12], information processing [16] or availability of attentional resources [35].

The duration and direction of gaze are highly related to what people see and understand about the visual world. An overt behavioral manifestation of selective attention is the place within a scene where viewers fixate their gaze, and the duration of such placement. Eye movements thus serve as a window into the operation of the attentional system [36]. Also, an increase in pupil diameter has been observed with increased attention [37, 38], cognitive control [39] and/or increased cognitive workload [35, 40]. There have been some attempts to measure pupil size during suprasecond time estimation tasks using the ‘time flies’ or ‘thinking aloud’ paradigms; these studies found that pupil diameter was larger during performance of the timed task (suggesting increased workload), and showed less variation than during non-timed tasks [41], and was negatively correlated with the expertise of the participants [42]. A study of eye movements during a thinking aloud task found brief fixations interspersed with frequent saccades between stimuli that were spatially far apart [43].

Based on the studies that showed that attentional mechanism affects timing in the subsecond scale (see above), our aim was to study eye movements and pupil diameter during a visual subsecond timing task; to our knowledge, there are no previous studies that accomplished this aim.

## Method

### Participants

The participants were 95 students from the Engineering Department of the University, aged 20–25 years (mean: 24.2 years); they were recruited by flyers. Exclusion criteria were a history of drug abuse or psychiatric or chronic medical illness during the last year, as reported by participants. From the 95 participants, 5 were excluded from analysis due to psychoactive drug usage in the last month; another 5 were excluded for tobacco or caffeine consumption in the last 3 h; all remaining participants were healthy and had a body mass index (BMI) between 18.5 and 25. Research participants provided written informed consent after they had received a written description of the experimental procedure. They were informed that the aim of the study was to examine the precision of their timing. The study was approved by the Ethics Committee of the Faculty of Psychology and conformed to the International Ethical Guidelines for Biomedical Research Involving Human Subjects (http://www.who.int/ethics/research/en/).

### Procedure of the bisection task

After a first telephone contact subjects were instructed to arrive at the lab after no sleep deprivation or somnolence. After their arrival at the lab they were seated in a light-controlled and sound-attenuated room, about 60–65 cm in front of a T120 Eye Tracker (Tobii Technology AB, Danderyd, Sweden). First, the eye tracker was calibrated for each subject by presenting 5 fixation points at the corners and center of the screen. Then, participants received oral and written instructions; in short, they were told that they should fixate their gaze on a cross (6 mm in width, located in the center of 4x3 cm rectangle with a black perimeter) for 100 msec (a successful fixation was indicated by the color of the perimeter of the rectangle changing from black to green). The computer screen was divided in 7 vertical by 7 horizontal rectangles measuring 3x5 cm, all of clear gray color numbered for 1 to 49 starting at the upper left and ending at the lower right. A colored (red, green, yellow, blue or magenta) circle 1 cm in diameter was presented at the square number 9, 13, 29, 39 or 41 for 200 or 800 msec and the subjects were instructed to press, as quickly as possible, the left key if the stimulus was “short” or the right key if the stimulus was “long”. Each correct response was rewarded with a point; total cumulated points were changed to monetary reward when the session ended. Stimulus presentation was programed with E-Prime (Psychology Software Tools Inc., Pittsburg, PA). Subjects received 5 trials to familiarize them with the fixation response and then 25 training trials in which they had only to fixate on the center cross and were rewarded with a smiling face when attained a 100 msec fixation. Thereafter, they had an 80 trial training session on which 200 and 800 msec stimuli alternated randomly between locations and color and subjects were rewarded with a point for each correct categorization of the duration of the stimulus. Each trial lasted for 2.5–3.0 sec (fixation time + stimulus duration + latency to respond) with a random intertrial time of 750–1500 msec. Then, subjects underwent a test session where 10 nor-reinforced stimuli of each intermediate duration (250, 320, 400, 500, 640 msec) were randomly intermixed with 15 reinforced and 5 non-reinforced trials (to be used for comparison with the intermediate durations) of each standard duration (200 or 800 msec).

### Eye-movement data preparation

The dependent variables were fixation position and pupil diameter of both eyes recorded at 50 Hz obtained with the E-Prime modules for Tobii. Only data from test trials were analyzed; however, when data indicated that direction of gaze was outside the screen and/or eye blinks occurred on more than 2 occasions within a trial, data from such trial were discarded (actually, no more than 2% of the data from any subject was discarded on these criteria). The area of the screen where each image was presented was defined as the Area of Interest (AoI), and fixation at those areas was defined when: 1) Saccades remained for at least 100 msec within one of the areas where stimuli were presented, 2) The initial saccade occurred more than 100 msec after stimulus onset (earlier fixations were considered anticipatory responses), and 3) Saccades that occurred more than 20 msec outside the AoI were considered as an independent saccade. The first analysis excluded data from trials when fixations did not meet these criteria.

### Data analysis

Data analysis and handling was done with Excel (Microsoft Corporation, Redmond, WA) and FileMaker Pro Advanced 11 (FileMaker, Inc. Santa Clara, CA). First, we obtained the latency to the first fixation, the duration of each fixation to any AoI, and the first AoI that was fixated or contacted was identified (in some cases, subjects made contact with an AoI but changed before 100 msec); then, trials were filtered to exclude those that do not fulfill the above-mentioned criteria. Trials in which the stimulus was presented at the center (20 out of 100) were not included, because there was no way to determine the latency since the subject might continue to fixate on the center after the preparatory fixation. Initially, the anchor, non-reinforced stimuli were considered separately, but since there were no differences to the anchor reinforced, all anchor trials were considered together. There was a wide between-subject variation in the proportion of trials that met the criteria; for some participants, more than 80% of the trials fulfilled the criteria, whereas for others less than 5% met the criteria. Therefore, we decided to study the extremes of the population: Two groups of 15 subjects were selected on the basis of the proportion of trials that met the criteria (>75% accepted and <5% accepted); 15 randomly selected subjects with intermediate accepted trials formed an additional group. For this analysis we included all the trials except those with more than 2 eye blinks or with fixations outside the screen. The analysis also determined the number of fixations at each AoI, the pupil diameter along each fixation and mean pupil diameter on each fixation and the latency and correctness of responses to stimuli of standard durations or categorization of stimuli as “short” (200 msec) or “long” (800 msec). The proportion of correct responses to the standard stimuli was used to determine discrimination index and a 3-parameter logistic function

$$f(x)=\left(\frac{\alpha }{1+{\left(\frac{x}{\beta }\right)}^{\varphi }}\right)$$

(where *alpha* is the asymptotic maximum, *beta* is the bisection point and *ph*i is the slope) was fitted to categorization data (proportion of “long” response to each intermediate duration) to estimate the bisection point (where subjects would choose a “long” response on 50% of trials), limen (range between 25 to 75 centile) and Weber fraction (ratio of bisection point to limen). One-way ANOVA was used to compare bisection points, limen and Weber Fractions between groups. Repeated measures two-way ANOVA was used to compare performance on other measures: discrimination index, latency, fixation duration or hits to AoIs. If significant results were obtained, *post hoc* Bonferroni`s test (significance criterion, p<0.05) was used to make comparisons between means with Prism (GraphPad Software, Inc. La Jolla, CA USA) or SPSS (IBM Corporation, Armonk, NY USA). Demographic and psychological test data were also analyzed but not included in the present paper.

## Results

As mentioned above, the data were filtered to identify those trials that fulfilled the inclusion criteria (latency ≥ 100 msec, duration ≥ 100 msec and contact with AoI where the stimulus was presented); it was found that for some subjects most trials were rejected while others had up to 95% of their trials accepted. Therefore, we selected two groups (n = 15) the extremes of the sample studied: those for whom less than 5% of trials were accepted and those for whom > 75% of trials were accepted. We selected a random sample of 15 subjects with an intermediate number of trials accepted; a preliminary analysis found no significant differences between data that included all trials to those that included only filtered trials for this sample of subjects. Therefore, we used all trials (excluding only blinking or saccades out of the screen) in further comparisons between the groups studied; otherwise, there would be no data from subjects that had their trials rejected by filtration criteria. As shown below, the subjects who had all their trials rejected maintained their gaze fixed on the central AoI (hence we name this group ‘central’, CNTR), while the subjects who had most of their trials accepted shifted their gaze towards the peripheral AoIs (hence we call this group ‘peripheral’, PRPH); the additional group in some trials maintained their gaze fixed on the central AoI, but in other trials shifted their gaze towards the peripheral AoIs (hence, we call this group “BOTH”).

### Discrimination performance

**Fig 1:**
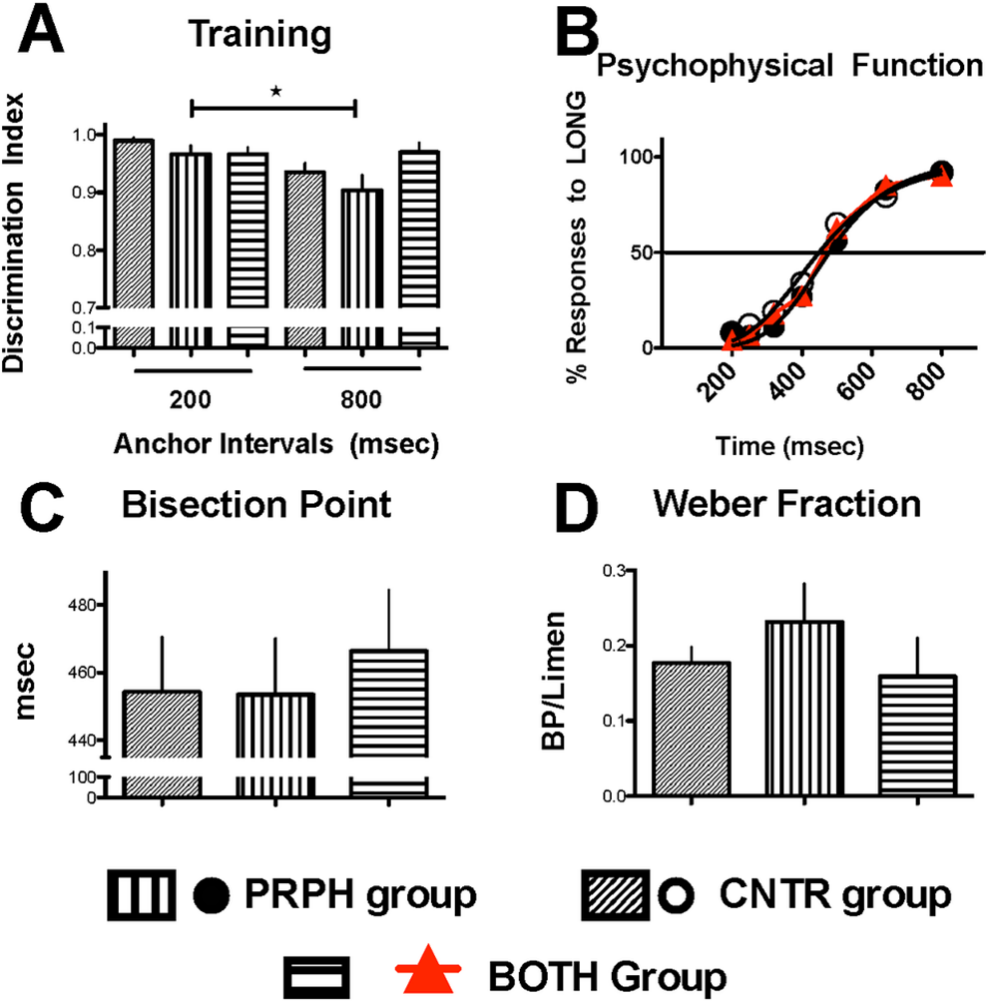
Timing performance on generalization test. (A) Discrimination index (Responses to 800 key/(Responses to 200 + Responses to 800 keys) maintained during the testing session. (B) Psychophysical function fitted to group data (N = 15 in each group) of responses to 800 msec key after intermediate durations. Bisection Point (C) and Weber fraction (D) derived from functions fitted the individual-subject data (see text). Each closed, open circle or red triangle and corresponding bars are means ± SEM (N = 15). In the PRPH group there was a significant difference between their discrimination indexes (see 1A)

Participants in all groups correctly identified stimulus duration as either “short” (200 msec) or “long” (800 sec), as indicated by their discrimination indexes which were >0.95 (Fig 1A). Discrimination indexes of subjects of the PRPH group tended to be smaller than those of the CNTR or BOTH groups. Two-way ANOVA (group x duration, with repeated measures on the latter factor) confirmed significant differences for duration (F(1,42) = 19.706, p = 0.037) and interaction (F(2,42) = 2.064, p = 0.004), but not for group (F(2,42) = 2.167, p = 0.127). *Post hoc* Bonferroni’s Test indicated that discrimination index for the 800 msec stimulus was significantly (p = 0.001) different from the 200 msec stimulus in the PRPH group. no other comparison attained statistical significance.

### Timing performance

The psychometric functions obtained from all groups are shown in Fig 1B. A logistic function was fitted to the data obtained from each subject to obtain estimates of the bisection point (Fig 1C), limen and Weber fraction (Fig 1D) in order to compare the groups’ performance. One-way ANOVA showed that there was no significant difference between the bisection points of the CNTR, PRPH and BOTH groups (F(2,44) = 0.179, p>0.05). The CNTR and BOTH groups tended to show lower and more homogeneous values of Weber Fraction than PRPH group (1D); however, one-way ANOVA indicated no significant difference between groups (F(2,44) = 0.768, p = 0.47).

### Fixation time

**Fig 2:**
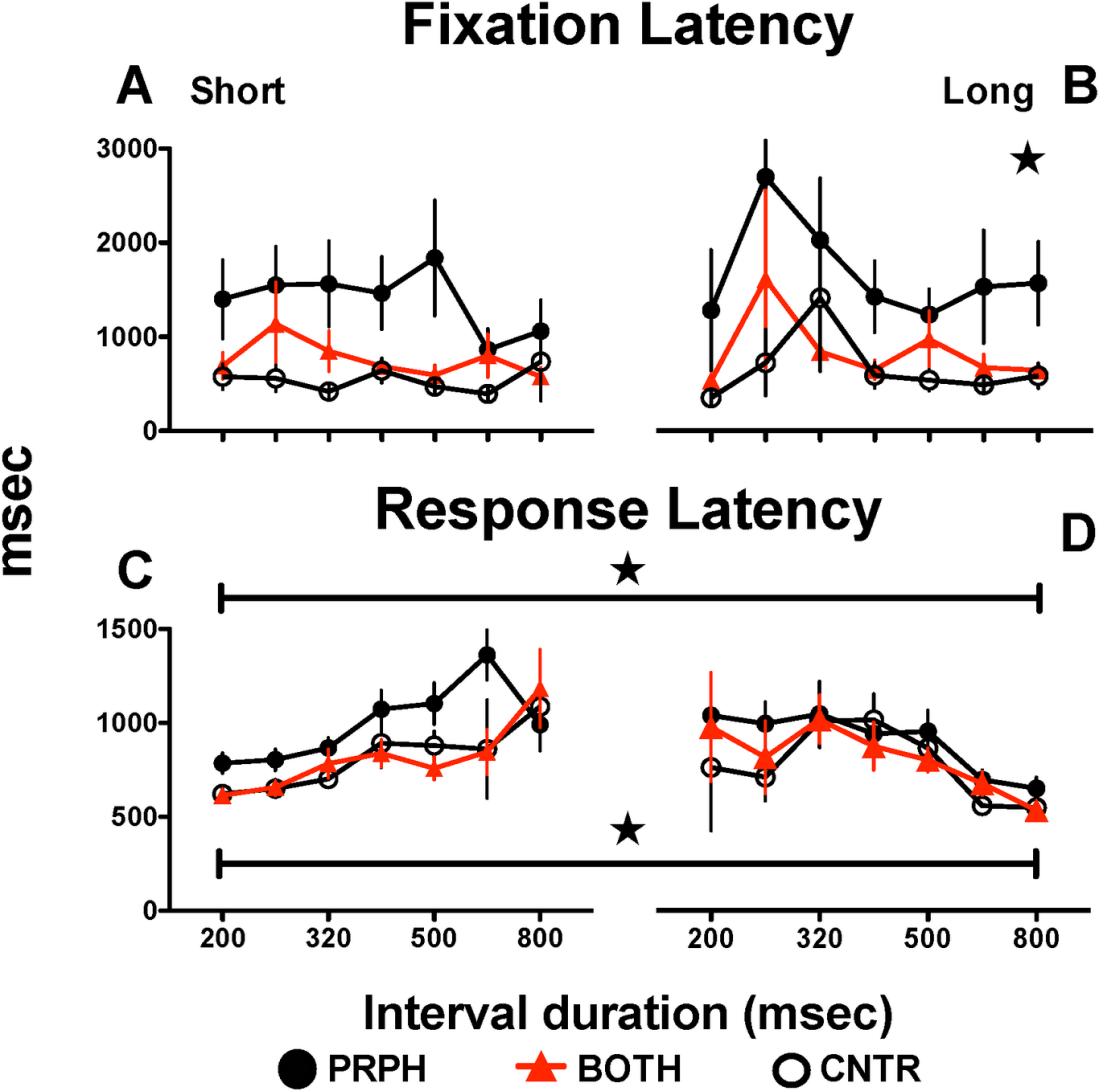
Fixation and response latency to “short” and “long” levers on generalization trials. Upper panels present latency to attain a 100 msec fixation on trials where subjects later responded to the 200 (A) or 800 (B) msec keys; lower panels present latency to emit categorization response of stimulus duration by responding to the 200 (C) or 800 (D) msec key. The performance of subjects (N = 15) of the CNTR group is represented by open circles while closed circles represent the performance of subjects (N = 15) of the PRPH group; the group that used BOTH is presented with red triangles. Only symbols at intervals close to or at the extreme durations present mean of 15 subjects since some subjects never emitted erroneous categorizations (e.g. response to 200 msec key after an 800 or larger than 400 msec stimulus). Stars and horizontal bars indicate significant differences between denoted groups after two-way ANOVA followed by Bonferroni´s test (p<0.05) (see text); only data from anchor intervals with N = 15 were included in statistical analysis.

At the start of each trial, subjects were required to fixate their gaze at the center of the screen in order to start a trial. Fig 2 shows the fixation time in trials when subjects chose to respond to “short” key (Fig 2A) or “long” key (Fig 2B). Each point indicates the latency that corresponded to the stimulus duration to be presented on the trial. ANOVA (group x stimulus duration) of the data obtained with the two anchor durations (200 and 800 msec) showed a significant difference between groups (F(2,42) = 3.631, p = 0.035), but not for stimulus durations (F (1,42) = 0.069, p = 0.794) or its interaction (F(2,42) = 0.638, p = 0.534). *Post hoc* Bonferroni’s test confirmed significant (p = 0.042) differences in fixation time between the PRPH and CNTR groups at 800 msec; no other comparison attained statistical significance.

### Latency to categorize durations as “short” or “long”

When the stimulus ended, subjects had to decide whether the preceding stimulus was similar to 200 or 800 msec by depressing the left or right key (respectively). Latencies to emit these responses are presented in Fig 2C (for responses to the “short” key) and Fig 2D (for responses to the “long” key). With stimulus durations of 640 or 800 msec subjects had short latencies to correctly categorize them as “long” (right panels); with durations of 200 to 320 msec subjects also had short latencies to categorize them as “short”. When subjects confronted difficult decisions (i.e. when they made a decision for a 400 msec stimulus, or made a mistake (choosing “short” when the stimuli duration was greater than 400 msec, or “long” when it was less than 400 msec)) latencies tended to be longer. However, as in the preceding case, the incidence of selection of “short” decreased as the stimulus duration increased (or vice versa in the case of “long”), precluding statistical comparisons for intermediate durations. Therefore, in this and subsequent comparisons, we compared only the correct extremes of the distributions where there were data from all subjects for the repeated measures ANOVA. Two-way ANOVA (group x stimulus duration, with repeated measures on the latter factor) indicated significant differences between latencies for the two stimulus durations (F(1,42) = 25.449, p<0.001), but no significant effect of group (F(2,42) = 2.917, p = 0.065) and no significant interaction (F(2,42) = 0.864, p = 0.429). *Post hoc* Bonferroni’s test confirmed that the latency after an 800 msec stimulus was significantly shorter than after a 200 msec stimulus for all groups (PRPH, p = 0.001; CNTR, p = 0.027, BOTH, p = 0.018).

### Fixation duration on each Area of Interest (AoI) during stimulus presentation

**Fig 3:**
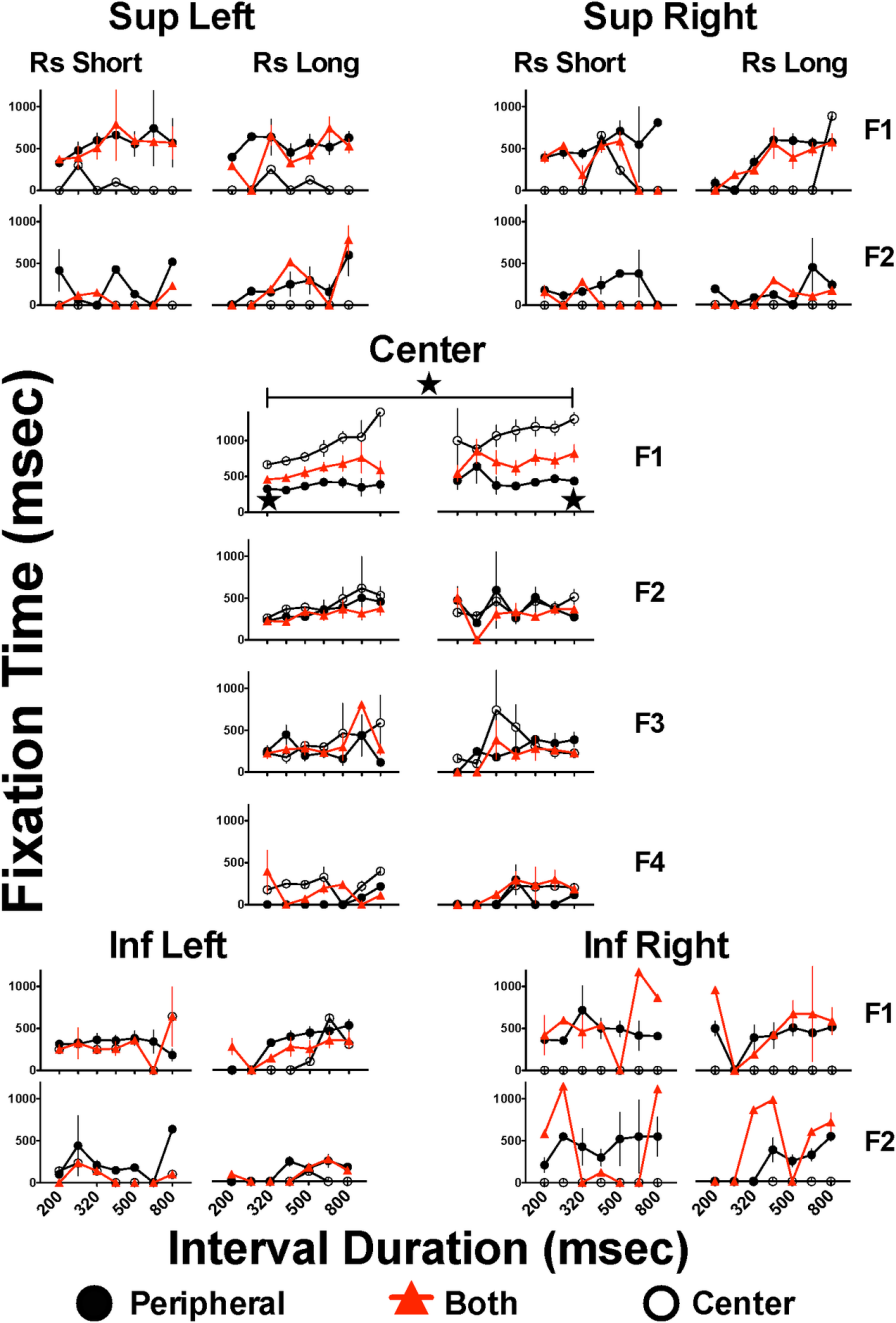
Duration of successive fixations on each Area of Interest during generalization trials. Mean fixation time in each successive fixation to each Area of Interest (AoI) where a stimulus could appear: fixation 1 (F1) to fixation 4 (F4) for Centre AoI but only F1 and F2 for remaining AoIs. For each fixation to each AoI, left panels present the performance on trials where subjects categorized intervals as “short” and right panels correspond to categorizations as “long”; only intervals close to or at the extreme durations present mean of 15 subjects since some subjects never emitted erroneous categorizations. Stars and horizontal bars indicate significant differences between denoted groups after two-way ANOVA followed by Bonferroni´s test (p<0.05) (see text); only data from anchor intervals with N = 15 were included in statistical analysis.

The cumulative duration of all fixations at each AoI revealed a clear difference between the two groups: the CNTR group cumulated fixation time by remaining at the central AoI, while the PRPH group cumulated fixation time at each AoI. The fixation time of the BOTH group was intermediate at the central AoI; on the occasion when these subjects gazed towards peripheral AoIs their cumulated fixation time tended to be similar to that of the PRPH group. Since the subjects could direct their gaze at the AoIs on several occasions during the stimulus presentation, we analyzed the average duration of each fixation. Fig 3 shows mean duration of the first 4 fixations (F1 to F4) to the central AoI and of 2 fixations (F1, F2) to the peripheral AoIs. Differences are readily visible: while the CNTR group made up to 4 fixations on the central AoI but seldom fixated on peripheral AoIs, the PRPH and BOTH groups made no more than 3 fixations on the central AoI but made up to 2 fixations on each peripheral AoI. In addition, the duration of the first fixation on the central AoI was longer in the CNTR than in the PRPH group. In the PRPH and BOTH groups the durations of fixations (when made) were similar for centrally directed and peripherally directed fixations, and did not differ between the first, second and third fixation. Furthermore, in the PRPH and BOTH groups, increasing the stimulus duration produced only a slight increment in fixation duration, whereas in the CNTR group fixation time was positively related to stimulus duration, in some cases exceeding the stimulus duration, suggesting that these subjects held their fixation on the central AoI not only for the duration of the stimulus but until they emitted their categorization response.

As in the preceding cases, the incidence of choosing “short” declined as the stimulus duration increased (and vice versa in case of “long”), which precluded statistical comparisons for intermediate durations; therefore, we compared only fixation duration when subjects responded on the “short” or “long” key when stimulus was 200 or 800 msec, respectively. Also, it was not possible to compare between successive fixations since not all the subjects made a second or third fixation to a particular AoI. Two-way ANOVA (group x stimulus duration) revealed significant main effects of duration (F(1,42) = 84.544, p<0.001) and group (F(2,43 = 19.391, p<0.001) and a significant interaction (F(2,42) = 22.405, p<0.001). The *post hoc* Bonferroni’s test confirmed that the fixation time to 800 msec stimuli was significantly longer than the fixation time to 200 msec stimuli in the CNTR and BOTH groups (p<0.001). Also, the fixation times to the 200 msec stimulus were significantly shorter in the PRPH (p<0.001) or BOTH (p<0.01) groups than in the CNTR group. In the case of the 800 msec stimulus the PRPH (p<0.001) and BOTH (p<0.002) fixations were shorter than that of the CNTR group.

### Pupil diameter during fixations

**Fig 4:**
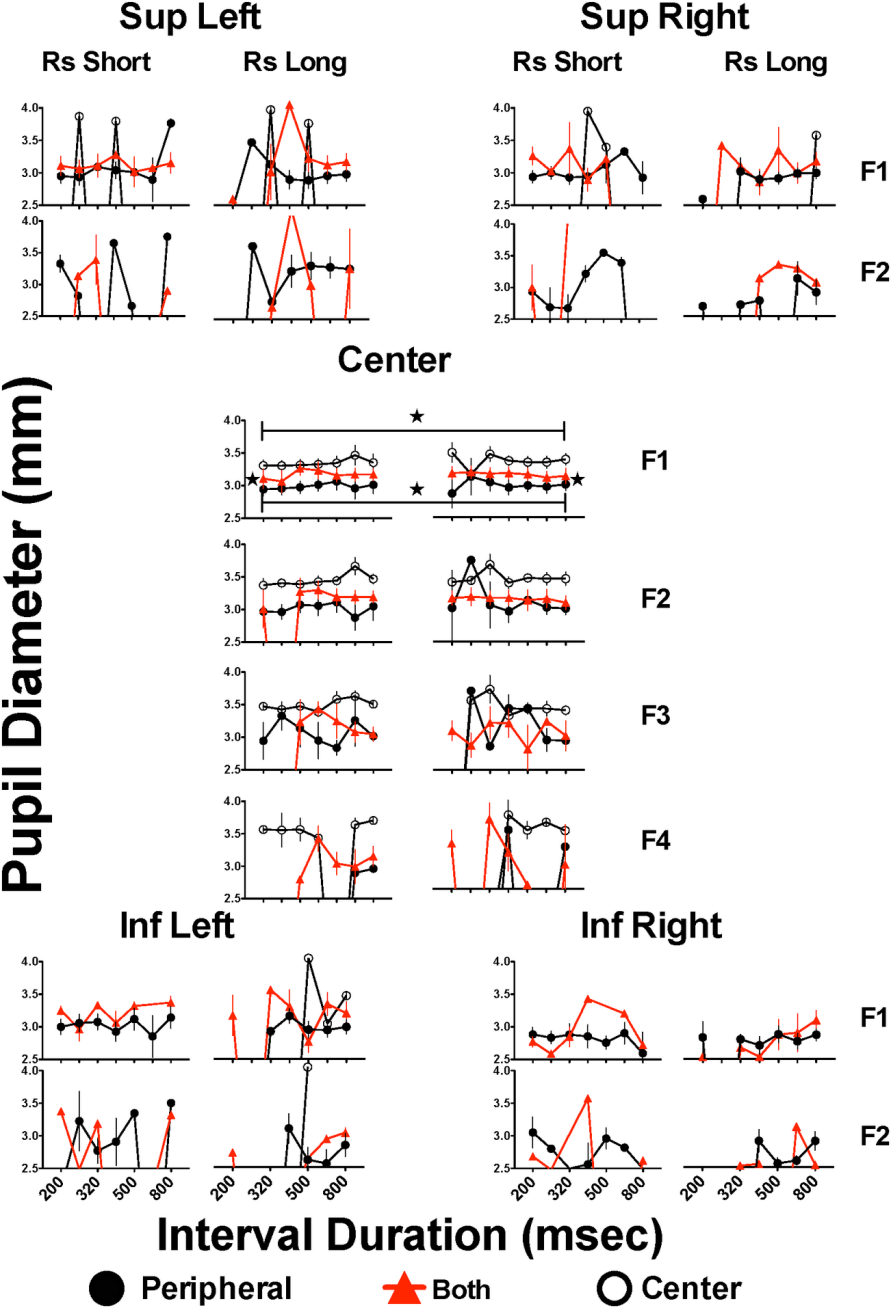
Mean pupil diameter in successive fixations on each Area of Interest during generalization trials. Mean pupil diameter in each successive fixation to each Area of Interest (AoI) where stimulus could appear: fixation 1 (F1) to fixation 4 (F4) for Centre AoI but only F1 and F2 for remaining AoIs. For each fixation to each AoI, left panels present the performance on trials where subjects categorized intervals as “short” and right panels correspond to categorizations as “long”; only intervals close to or at the extreme durations present mean of 15 subjects since some subjects never emitted erroneous categorizations. Stars and horizontal bars indicate significant differences between denoted groups after two-way ANOVA followed by Bonferroni´s test (p<0.05) (see text); only data from anchor intervals with N = 15 were included in statistical analysis.

Fig 4 shows pupil diameter during each fixation. Pupil diameter tended to be larger in the CNTR than in the PRPH group; also, the diameter was greater in the case of difficult classifications (close to 400 msec) or when subjects emitted inconsistent responses (i.e. choosing “short” when the stimulus was longer than 400 msec or “long” when the stimulus was shorter than 400 msec). Two-way ANOVA (group x stimulus duration) revealed significant main effects of stimulus duration (F(1,42) = 18.655, p<0.001) and group (F(2,42) = 4.048, p = 0.025), but no significant interaction (F(2,42) = 1.574, p = 0.219). The *post hoc* Bonferroni’s test confirmed that the pupil diameter was smaller in the PRPH than in the CNTR group when subjects were confronted with stimuli of 200 (p = 0.024) or 800 msec (p = 0.019). Also, the pupil diameter was larger when confronted with 800 than with 200 msec stimulus in both the PRPH (0.005) and the CNTR (p = 0.001) groups.

### Number of valid fixations (duration and latency larger than 100 msec)

**Fig 5:**
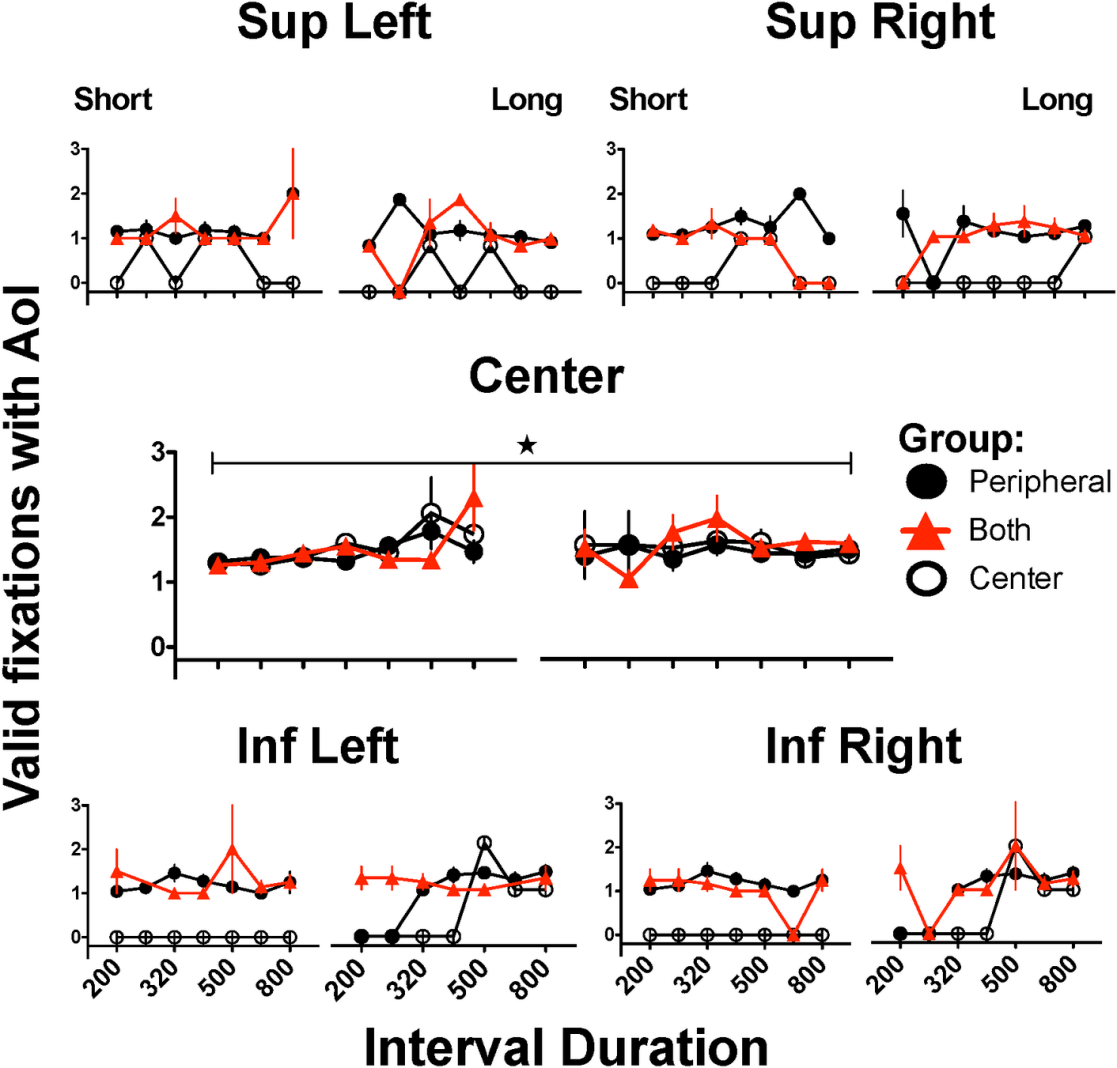
Valid fixations to each Area of Interest during generalization trials. Valid fixation to each Area of Interest (AoI) where stimulus could appear. For each AoI, left panels present the performance on trials where subjects categorized intervals as “short” and right panels correspond to categorizations as “long”; only intervals close to or at the extreme durations present mean of 15 subjects since some subjects never emitted erroneous categorizations. Stars and horizontal bars indicate significant differences between denoted groups after two-way ANOVA followed by Bonferroni´s test (p<0.05) (see text); only data from anchor intervals with N = 15 were included in statistical analysis.

We considered the possibility that the rejection of trials was related to the stringent criteria; therefore, we counted fixations that fulfilled the initial filtration criteria (at least 100 msec duration and latency larger than 100 msec in the case of peripheral AoIs). As shown in Fig 5, while the PRPH or BOOT groups made 100 msec or longer fixations to all the AoIs, the CNTR group made fixations only to the central AoI. Comparing the groups’ fixations on the central AoI during presentation of the 200 and 800 msec stimuli (when subjects responded to “short” or “long” keys, respectively), two-way ANOVA (group x stimulus duration) showed a significant main effect of stimulus duration (F(1,42) = 22.434, p = 0.001), but not of group (F(2,42) = 1.751, p = 0.186), and there was no significant interaction (F(2,42) = 1.794, p = 0.179). The *post hoc* Bonferroni’s test found only marginal differences for the number of valid fixations in the PRPH and BOTH groups when subjects were confronted with stimuli of 200 or 800 msec (p = 0.001 and p = 0.005 respectively). None of the other comparisons attained statistical significance.

### Number of fixations to all AoIs irrespective of latency or duration

**Fig 6:**
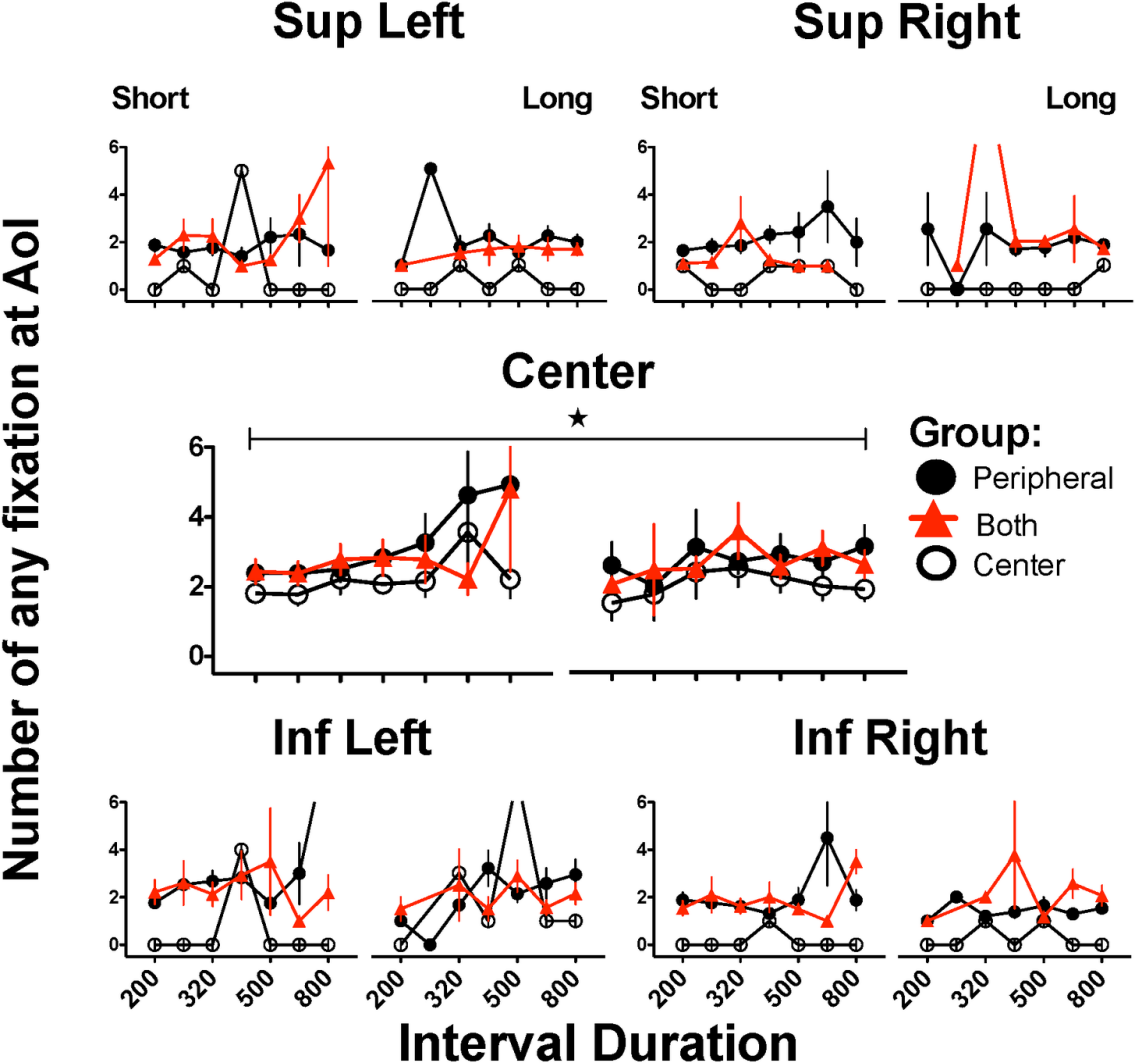
All fixations to each Area of Interest during generalization trials. Number of any fixation (includes fixations even if duration and latency criteria were not meet) to each Area of Interest (AoI) where a stimulus could appear. For each AoI, left panels present the performance on trials where subjects categorized intervals as “short” and right panels correspond to categorizations as “long”; only intervals close to or at the extreme durations present mean of 15 subjects since some subjects never emitted erroneous categorizations. Stars and horizontal bars indicate significant differences between denoted groups after two-way ANOVA followed by Bonferroni´s test (p<0.05) (see text); only data from anchor intervals with N = 15 were included in statistical analysis.

To further explore if the rejection was related to stringent criteria, we eliminated any criteria (latency or duration) and counted the fixations to all AoIs. As shown in Fig 6, the PRPH and BOTH groups made, on average, 2 fixations to each AoI. It is also apparent that, as the stimulus duration increased, subjects in the PRPH group made more fixations to the AoIs, whereas the CNTR group consistently made, on average, 2 fixations to the central AoI, but very few fixations to peripheral AoIs; on such rare occasions these fixations were too short or too early to fulfill the initial criteria, as suggested by comparison of this figure with the preceding one. Peaks on fixation number at peripheral AoIs are of very few subjects that made a fixation. It is not apparent that subjects made more fixations to the superior or right AoIs (see discussion). Two-way ANOVA (group x stimulus duration) showed a significant main effect of stimulus duration (F(1,42) = 5.996, p = 0.019), but not of group (F(2,42) = 1.581, p = 0.218), and no significant interaction (F(2,42) = 2.226, p = 0.121). The *post hoc* Bonferroni’s test found a smaller number of fixations in the PRPH group when subjects were confronted with stimuli of 200 msec than when confronted with 800 msec stimuli (p = 0.003). No other comparisons yielded statistical significance.

### Number of fixations to wider peripheral AoIs irrespective of latency or duration

**Fig 7:**
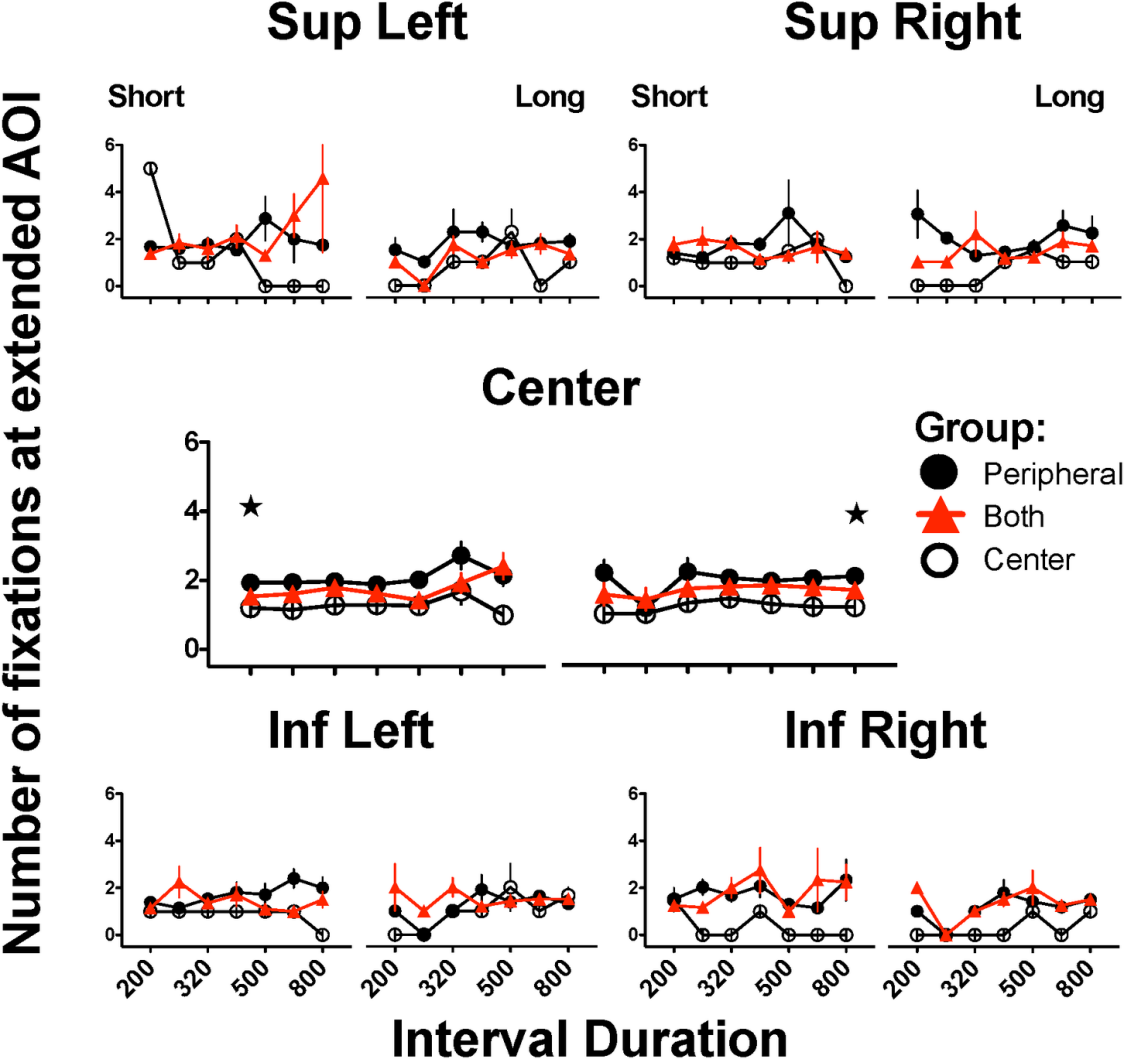
Fixations to extended Areas of Interest during generalization trials. Number of fixations to redefined (expanded) Area of Interest (AoI) where a stimulus could appear. For each AoI, left panels present the performance on trials where subjects categorized intervals as “short” and right panels correspond to categorizations as “long”; only intervals close to or at the extreme durations present mean of 15 subjects since some subjects never emitted erroneous categorizations. Stars and horizontal bars indicate significant differences between denoted groups after two-way ANOVA followed by Bonferroni´s test (p<0.05) (see text); only data from anchor intervals with N = 15 were included in statistical analysis.

Finally, we examined whether the subjects of the CNTR group made eye movements in the direction of the peripheral AoIs that were too short to hit the AoI where the stimulus was located. To this end, we redefined the AoIs to include a wider area around each AoI and then counted the hits to those “extended” AoIs. As mentioned in the Procedure section, the screen was divided in 7x7 areas, and Superior Left AoI was defined to be 9 and then redefined to be 2, 8, 9, 10, 16, 17; Superior Right to be 6,12,13, 14,19 and 20; Inferior Left: 30, 31, 36, 37, 38 and 44 and Inferior Right: 33, 34, 40, 41, 42, 48. The central AoI was redefined to be 18, 24, 25, 26 and 32. This redefinition had some impact on the data from the two groups since with the new definition small saccades away from an AoI (i.e., saccades that did not exit the extended area) were counted as belonging to the same fixation (seen mainly in the PRPH group). Moreover, a saccade that was too short to reach a peripheral AoI under the original criteria, was now counted as a fixation (seen mainly in the CNTR group). Thus, while similar data were observed in the PRPH group, a clear difference emerged for the CNTR group between the two figures. Fig 7 shows that the CNTR group hit the extended areas on more occasions than in Fig 6, the explanation for the difference being that saccades that were too short to be detected in the former analysis emerged with the present analysis); with the expanded AoIs, performance of BOTH group was in between the extremes. Two-way ANOVA (group x stimulus duration) yielded a significant main effect of group (F(2,42) = 10.686, p = 0.001) and stimulus duration (F(1,42) = 4.203, p<0.047); but there was no significant interaction (F(2,42) = 1.284, p = 0.288). The *post hoc* Bonferroni’s test revealed a larger number of hits to the central AoI fixations in the PRPH group when subjects were confronted with stimuli of 200 or 800 msec than those of the CNTR group (both p<0.001); the PRPH group also made more fixations than the BOTH (p<0.037) group when confronted with a stimulus duration of 200 msec. No other comparisons attained statistical significance.

## Discussion

The subjects learned the time discrimination task in only one training session of 80 trials and were able to maintain their correct discrimination in at least 95% of the 200 or 800 msec trials of the test session (despite 20% of these trials being unreinforced). Also, subjects were able to categorize the stimulus durations as “short” or “long” (bisection task) when intermediate durations were introduced (see below). Some differences between subjects became apparent after using filtering criteria similar to those used in dot probe tasks [44, 45]. First, fixations were required to be longer than 100 msec toward the area where the stimulus was presented (Area of Interest, AoI); the purpose of this criterion was to exclude saccades aimed at another location that by chance crossed the actual AoI [46]. Second, fixation latencies shorter than 100 msec were considered as premature responses, meaning that the fixation coincided by chance with the actual location of the stimulus. When we applied these criteria to the filtering process, we excluded all trials (20 trials) in which the stimulus appeared at the central AoI, since it was not possible to determine an anticipated gaze towards the area that was also used as the fixation point. After filtering, two sets of subjects emerged: one that held their gaze at the central AoI (CNTR), and the other that directed their gaze at peripheral AoIs (PRPH); we also included a group that had an intermediate number of trials accepted (BOTH). To further compare the performance of subjects, we considered all trials (excluding those trials with eye blinks, those where the gaze was outside the screen and those that had the stimulus at the central AoI) to compare groups.

When subjects were confronted with intermediate durations and their percentage of “long” responses was individually fitted with the logistic function to generate a psychometric function, their bisection points (BP) were close to the geometric mean of the trained durations and were similar to those reported by others who used similar training durations and logarithmic distribution of intermediate durations (probe of 600 msec [47], 200 vs 800, BP of 462 [48], 300 vs 900, BP of 610 [49]); also, the observed Weber Fraction was within the range reported by these authors. Of interest, no significant differences were observed in the bisection point between groups, suggesting that all groups achieved a similar timing performance despite they used different attentional mechanisms (see bellow).

Earlier formulations of timing models [7, 17, 50–53] suggested several mechanisms on which the timing process could rely, but these did not include attentional processes. In later revisions of these models, attentional processes were included, which were assumed to increase the pacemaker rate (arousal), affect the gate or switch, the memory stages, or the combination of gate and memory [54, 55]. Other models in the cognitive tradition included attentional mechanisms from their inception; in these models attention was also assumed to modulate arousal [16], gate or switch [18, 20, 35] or memory stages [56]. In the context of the SET model, Lejeune (33) suggested that if the subject was highly attentive, switch operation would closely follow stimulus onset and offset, resulting in minimal variance of the pacemaker pulses; however, if the subject was paying little attention to a stimulus, its offset and onset might be “blurred”, resulting in greater switch variance. Using different models others confirmed that alterations in attention increased the variance of estimations [5, 19–21, 57]. In our case, although no statistically significant differences emerged between groups, the CNTR and BOTH groups had a lower Weber Fraction and their discrimination index was slightly larger when compared to the PRPH group; this may suggest better performance since an increased variability (as revealed by a shallower slope of the psychophysical function) has been interpreted as attentional in origin in the framework of pacemaker clock models. However, the most remarkable finding is that this minor alteration in variability is insufficient to produce an alteration in the bisection point.

Most previous studies of attention allocation focused on explaining the process that determines fixation location rather than its duration [58]; models that also tried to predict duration suggested that fixation duration is extended (saccade cancelation) when processing complex information (e.g. [59]). The fovea makes up only a tiny portion of the visual field, but foveal processing is invoked during fixations when there is need of fine-detail discrimination [50, 60]. The large concentration of rods at extrafoveal area allows greatest illumination sensitivity and faster conduction rates, so extrafoveal information is used to obtain global image characteristics and saccade guidance [60, 61]; in order to direct eye movements efficiently, the visual system must integrate low-resolution information in the visual periphery with knowledge about the current task and environment [62]. In the present experiment each AoI had a dimension of 3.86 x 4.93 cm; at 60 cm from the screen, this converts to an AoI of 3.68 x 4.7 degrees of the visual field. From the fixation point to any AoI subjects needed to produce a saccade of 5.97 degrees; therefore, subjects could use the extrafoveal mechanism to detect disappearance of the stimulus even with short saccades. Since instructions to subjects did not mention whether they had to identify a particular characteristic of the stimulus, it was possible to use of extrafoveal mechanism to detect a change in illumination of the AoI to determine the presence or absence of the stimulus; this strategy, which seems to have been favored by the CNTR (and used in some occasions by the BOTH) group, may have been responsibly for generating the lower Weber Fraction in these groups.

Two general theoretical models of visual attention allocation have been suggested: bottom-up (also referred to as stimulus-driven, automatic, or exogenous orienting), versus top-down (also referred as goal-directed, controlled, voluntary, endogenous, or based on cognitive structures of knowledge) [29, 35, 63, 64]. The visual saliency hypothesis, which proposes that the information generated by the image drives the allocation of visual attention and thus the placement of fixations in a scene, favors the bottom-up stimulus-based models [65]. The cognitive control hypothesis, which proposes an unprioritized input representation, favors a top-down guidance of attention [66, 67]. In bottom-up models, the emphasis is on the focus of attention being involuntary but driven by an inherently salient or transient exogenous orienting stimulus; as attention is a reaction to the visual properties of the stimulus confronted by the viewer, it is less susceptible to other forms of cognitive interference. In top-down models, the emphasis is on voluntary, intentional endogenous orienting attention to a specific location, usually guided by a symbolic cue; eye movements are primarily controlled by task goals interacting with a semantic interpretation of the scene and memory traces of similar episodes, and is quite susceptible to cognitive interference [59, 65]. Another common finding is that exogenous cues are harder to ignore and induce faster and more transient effects than endogenous orienting attention [68]. In this study, the filtering processes gave evidence of the existence of two cognitive strategies: one group chose to retain their gaze at the central position of the screen while the other group gazed toward peripheral locations while deciding whether the stimulus had disappeared, in order to categorize it as “short” or “long”; there is another group of subjects that in some trials used one or the other strategy. These strategies seem similar to the top-down and bottom-up (respectively) strategies previously described, and are clearly seen in Fig 3 (above). The stimulus to be timed was displayed immediately after a fixation of 100 msec at the central AoI; for the CNTR group (and on some trials of the BOTH group), instead of considering the latency as a waiting time to emit a valid fixation to the stimulus AoI, it is more precise to say that subjects held their gaze at the central AoI for the duration of the stimulus until they emitted a categorization response. On a few occasions, they left the central position (see F2 to F4 in Fig 3 and Fig 5) but quickly returned to the central AoI. When we used a wider definition of an AoI (as in Fig 7) the number of hits of CNTR subjects to the central AoI was reduced because they produced short saccades, too close to the (previously defined) central AoI that in consequence were included as a continuation of the previous fixation to the central AoI in the wider definition. The preferential use of one of these two attention processes may also explain the easiness of the CNTR and BOTH groups to attain the initial fixation: the central control of their saccades allowed them to easily hold their gaze for 100 msec within 600–700 msec and with minimum variability, while the PRPH group took more than 1.2 s and had larger variability to achieve a 100 msec fixation.

The average fixation duration during scene viewing has been reported to be 300 [69], 330 [67] or within the range of 50–1000 [70] msec, despite considerable variability in fixation location. A relatively recent model of eye movements [59] assumes that saccade duration is generated by a random sampling of a duration distribution; if there is a difficulty at the level of visual or cognitive processing, then the next saccade initiation is inhibited (saccade cancelation), leading to a longer fixation to allow acquisition of visual information [71]. Saccade cancelation by a stimulus-based mechanisms has been considered as evidence for a stimulus-driven selection (bottom-up) mechanism that supersedes observers’ cognitive (top-down) control of gaze [67]. An extrafoveal stimulus may not be fully analyzed before it is fixated, but partial analysis of it provides information that subsequently speeds its analysis once it is fixated [72]. In real-world scene search tasks the first saccade tends to land near regions that are likely to contain the target [62, 73] than on areas with salient targets [66]. It has been suggested that the duration of the first fixation mainly reflects object identification while the mean gaze duration reflects post-identification processes such as memory integration [74]. In our case, duration of the first saccade was larger in the CNTR group, intermediate in the BOTH group and shorter the PRPH group, but rather than being engaged on an identification process we suggest that subjects in the CNTR group were actively canceling the following saccade, waiting for illumination change to determine stimulus offset. When we compared cumulated fixation time across all AoIs for the PRPH and CNTR groups (see S1 Fig) we observed that the cumulated time for the PRPH group was significantly longer than for the CNTR group at the anchor durations, suggesting that the strategy used by the CNTR group was more efficient than that used by PRPH group in order to get a decision, without affecting the correct estimation of time.

An analysis of sequences of hits to AoIs during the saccade indicated that subjects hit a peripheral AoI and immediately returned to the central AoI; on very rare occasions they moved from one to another peripheral AoI. As a consequence and since longer saccades or more fixations also meant longer times, the PRPH group made fewer valid hits to the central AoI (see F2 to F4 in Fig 3). However, Figs 6 and 7 suggest that as time passed, short saccades increased (see columns for 500 and 640 intermediate stimuli in both figures). In the case of the CNTR group the analysis of the sequence of hits to AoIs gave similar results: subjects made a saccade toward a peripheral AoI and immediately returned to the central AoI instead of going to another peripheral AoI; but in this case, saccades were too short to reach the peripheral AoIs. Performance of the BOTH group was intermediate to the two other groups. Although saccades may be an adjunctive (meditational) behavior used to estimate elapsed time [33, 75], their execution may also compete for central resources and represent a larger load to the attentional mechanism and, therefore, their execution may reduce sensitivity to time and explain the larger (although not statistically different) Weber Fraction of the PRPH group.

An asymmetry between short and long categorizations in the temporal bisection task has been described previously: it has been reported that response times to categorize “long” stimuli are shorter than when categorizing “short” stimuli and the categorization of short but not long stimuli are modulated by the probability of the reference durations [76]. At the moment when the categorization response is emitted, subjects are assumed to perform an assessment of the involved risk of misclassification; during long stimuli, once a criterion (above the indifference point) is attained, the “long” response is favored and subjects commit thereon to that response (producing a time gain of motor preparedness) even before the end of the stimulus and do not rely (as in the case of short stimuli) on post-stimulus decisions regarding the differences in duration [76]. In a similar way, rats [77] and pigeons [78] move from the location associated with the “short” operandum to the location associated with the “long” operandum when the stimulus duration approaches the point of subjective equality.

Coskun, Sayali (76) found that reactions times to emit a response were faster for correct compared to erroneous categorizations. In the present experiment in all groups, latencies to correctly categorize stimulus as “long” were short when compared to the correct categorization of stimulus as “short”. It is noteworthy that the longest latencies are observed with stimuli close to the bisection point and in the direction of a wrong response (i.e. categorizing a stimulus as “long” when it was short or “short” when it was long). Also of relevance, long latencies correlated with longer fixations (in the CNTR group) or an increased number of fixations to peripheral AoIs and longer cumulated fixation time observable in the PRPH group. Difficult categorizations presumably require more processing time to reach a decision. Minimum reaction times are observed when subjects had to press “short” with the left hand and “long” with the right [76, 79], according to the proposed cognitive representation of a time line [80] or mental magnitude line [26, 81, 82]. In our case, the “short” key was on the left-hand side of the keyboard and “long” on the right; this could have shortened latencies, although subjects did not receive special instruction for using the left or right hand to respond. Also, we wondered whether the time line or mental magnitude line could induce an increase in saccades toward the superior or right AoIs, but there is no evidence of such effect on Figs 5 to 7.

Pupil size is dependent on the luminance of the display. In addition, pupil diameter may be distorted by the subject's gaze angle when using head-mounted or desktop cameras coupled to eye-trackers. However, distortions are minimized when using tracker systems (like the Tobii 1750) that use the length of the major axis of an ellipse fitted to the pupil image to obtain a reliable measure of pupil size [83]. Changes in pupil diameter track preconscious or automatic processing and accompanying violations of expectations [38]. Pupil diameter is regarded as a good measure of attention or cognitive load, since as task difficulty, cognitive workload, and/or arousal increases, performance gradually degrades, producing a concomitant increase in baseline diameter [84–88]. Indeed, a neural model has been proposed [89, 90] which relates Locus Coeruleus function and pupil diameter [91] with attention [39] and cognitive processing [37]. Traditionally it has been considered that pupil size increases slowly in response to a relevant event and peaks after approximately 1 s, therefore, measuring effort by assessing pupil dilatation has been reserved for long or slow tasks. However, pupil diameter has been used recently (after deconvolution analysis) to document attention during a task that presented stimuli at a high rate [92] or when detecting a visual target during a rapid serial visual presentation [38, 56].

During performance of a timing task under the “time flies” paradigm (in the supra-second range) pupil diameter was larger (suggesting increased workload) and had less variation than during the execution of non-timed tasks [40]; also, minimum pupil diameter was larger and maximum pupil diameter smaller at the end of solved rather than unsolved tasks, suggesting less variation in mental workload during solved tasks [41]. Changes in pupil diameter observed in this study are consistent with these findings: pupil diameter was minimal when decision corresponded to a “long” response after a stimulus of 800 msec, intermediate for categorization of “short” after a 200 msec stimulus, and largest close to the bisection point or when subjects made wrong categorizations. These results suggest that long latencies, increased number of fixations per trial, or larger pupil diameter predict wrong categorizations.

As mentioned above, several authors [8, 9, 14, 36] have suggested that processing sub-second intervals is sensory-dependent and should not primarily depend on working memory and attentional allocation abilities, nor on motivational aspects of the task, while temporal processing of time intervals longer than 1 s requires the support of cognitive resources. Therefore, two different systems of temporal processing have been suggested: a more “automatic” one used to time in the millisecond range shared with motor coordination [24, 47], and a more “cognitive” one used for time estimation or reproduction and memory functions in the seconds to minutes range [93, 94]. Since it has been impossible to establish precise boundaries between the two temporal systems, Karmarkar and Buonomano (50) suggested that these systems may overlap at intermediary ranges (400–800 msec) and both mechanism may be used to time intervals in this range. However, Burle and Casini (20) and Lake, LaBar, and Meck [27], using timing tasks with intervals in the subsecond range, observed differences in the Weber Fraction consistent with attentional effects in the subsecond range. The present results, based on recording of eye movements and pupil dilatation, provide further evidence that the estimation of time intervals in the subsecond scale is not affected by the use different (or a mixture of) attentional mechanisms.

The executive-gate model [56] that evolved from the attentional-gate model [5] tried to explain prospective time judgments and suggested that attentional mechanisms may affect the gate or the switch. The relationship between these two constructs was explicated by Block and Zackay [21], “…we are unsure about the relative location of two components, the attentional gate and the switch…It may be more appropriate to locate the switch before, instead of after the attentional gate. Neither logical analysis nor empirical evidence seems to favor one order over the other”. Posner and Petersen (10) argued that, the switch operates as the result of the demands of external events while the gate operates as a result of the organism’s allocation of attentional resources; therefore, the switch implies a bottom-up control of attention while the gate a kind of top-down control of resources. Our data suggest that subject may use either or both strategies: some selected the top-down (keep fixation at the central AoI), others selected the bottom-up (follow the stimulus onset-offset) strategy, while others used both, leading to an intermediate number of trials being accepted with the criteria mentioned above. Further research is needed to determine the locus of attentional modulation; however, present results suggest that if the filtering processes (gate or switch) is maintained constant a minimal effect is produced on the accumulator processes

Irrespective of the specific details of the implementation of the timing mechanism, when execution divides attentional resources between external stimuli and attention to time, or requires that attentional or memory resources be shared by different tasks, the estimated durations should be shorter on prospective paradigms. There is substantial evidence that sharing attentional resources with a non-temporal task shortens subjectively perceived time (interference effect); this evidence derives from various paradigms, including the “thinking aloud” [23] and “time flies” [95, 96] paradigms, and many instances of the interference effect mentioned above. Also, conditions where attentional processes are compromised, such as the modulation of time estimation by the emotional content or context of the stimulus to be timed [97, 98], and the observation of increased time variability in subjects with Attention Deficit Hyperactivity Disorder [99, 100], support the notion that attention modulates timing in the suprasecond range. In further experiments, we expect that using a task on which subjects have to report color or form of the stimulus in some trials while in other trials they have to report if stimulus is “short” or “long” (as used by [69], [101]) should favor the use of the bottom-up or stimulus-driven mechanism and maybe produce decrements in time estimation in the subsecond range.

In conclusion, the present results provide evidence that during a timing task subjects may use a bottom-up, a top-down or a mixture of both mechanisms for scanning changes in the image projected on the retina. However, these strategies produced no differences in the bisection point, although such strategies were able to affect the variance of timing performance, the time to attain a fixation, the response latency, and the pupil diameter. Further work should determine whether the selection of attentional mechanisms represents idiosyncratic cognitive “styles” or is related to particular circumstances (task requirements or instructions) that modulate the attentional mechanism used by subjects.

## Supporting Information

S1 Fig Fixation time on a Trial. Figure shows mean cumulated fixation time on a trial independent of Area of Interest gazed.
(PDF)

S1 Table Spreadsheet with data. Each Sheet corresponds to one figure.
(XLS)
