# Supplementary figures and images for: Attentional Mechanisms during the Performance of a Subsecond Timing Task
Source: PLoS One. 2016 Jul 28;11(7):e0158508. doi: 10.1371/journal.pone.0158508 (PMC4965134; doi:10.1371/journal.pone.0158508)

Cumulated time during a fixation to any interval (msec).

## Total fixation time on a Trial

### Short

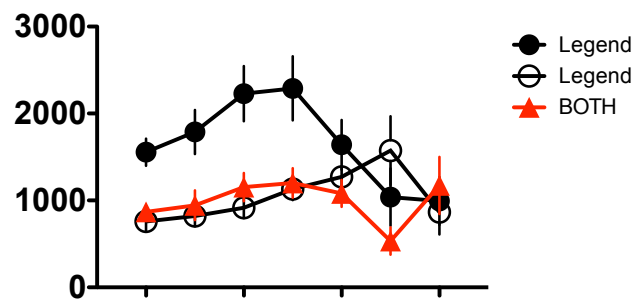

### Long

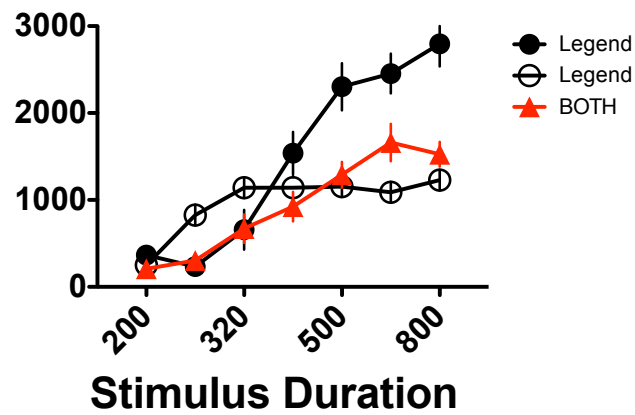

Supplement: S1 Fig — Figure shows mean cumulated fixation time on a trial independent of Area of Interest gazed. (PDF) [file pone.0158508.s001.pdf]
